# Inequalities in health: definitions, concepts, and theories

**DOI:** 10.3402/gha.v8.27106

**Published:** 2015-06-24

**Authors:** Mariana C. Arcaya, Alyssa L. Arcaya, S. V. Subramanian

**Affiliations:** 1 Department of Social and Behavioral Sciences, Harvard T.H. Chan School of Public Health, University in Boston, MA, USA; 2 Region 2, United States Environmental Protection Agency, New York, NY, USA

**Keywords:** health disparities, inequality, inequity, theory

## Abstract

Individuals from different backgrounds, social groups, and countries enjoy different levels of health. This article defines and distinguishes between unavoidable health inequalities and unjust and preventable health inequities. We describe the dimensions along which health inequalities are commonly examined, including across the global population, between countries or states, and within geographies, by socially relevant groupings such as race/ethnicity, gender, education, caste, income, occupation, and more. Different theories attempt to explain group-level differences in health, including psychosocial, material deprivation, health behavior, environmental, and selection explanations. Concepts of relative versus absolute; dose–response versus threshold; composition versus context; place versus space; the life course perspective on health; causal pathways to health; conditional health effects; and group-level versus individual differences are vital in understanding health inequalities. We close by reflecting on what conditions make health inequalities unjust, and to consider the merits of policies that prioritize the elimination of health disparities versus those that focus on raising the overall standard of health in a population.

Policymakers, researchers, and public health practitioners have long sought not only to improve overall population health but also to reduce or eliminate differences in health based on geography, race/ethnicity, socioeconomic status (SES), and other social factors (e.g. 1, 2). This paper aims to create a centralized resource for understanding methodological, theoretical, and philosophical aspects of health inequalities research in order to help advance health inequalities research. It synthesizes and expands upon previously published work that addresses concepts relevant to the study of health inequalities and inequities (3–7). The article begins by clarifying vocabulary needed to describe differences in health, whether they are observed across places and social groups, or among individuals in a single population. Next, it introduces key concepts for gathering and interpreting information on health inequalities. It considers the ways in which researchers and policymakers explore health inequalities, including by social groups, or by geographic area. The article then provides an overview of theories commonly employed to explain health differences. Finally, we conclude by considering ethical questions raised by health disparities and questions policymakers might consider when structuring programs and policies to address health disparities.

## Motivation for studying health inequalities

Despite considerable attention to the problem of health inequalities since the 1980s (8), striking differences in health still exist among and within countries today (9). In 2010, for example, Haitian men had a healthy life expectancy (10) of 27.8 years, while men in Japan could expect 70.6 years, over twice as long, in full health (11). Social group differences within countries are also often substantial. In India, for example, individuals from the poorest quintile of families are 86% more likely to die than are those from the wealthiest fifth of families, even after accounting for the influence of age, gender, and other factors likely to influence the risk of death (12). When health differences such as these are observed, a primary question of interest is whether the inequality in question is also inequitable.

### Health inequalities versus health inequities

The term *health inequality* generically refers to differences in the health of individuals or groups (3). Any measurable aspect of health that varies across individuals or according to socially relevant groupings can be called a health inequality. Absent from the definition of health inequality is any moral judgment on whether observed differences are fair or just.

In contrast, a *health inequity*, or health disparity, is a specific type of health inequality that denotes an unjust difference in health. By one common definition, when health differences are preventable and unnecessary, allowing them to persist is unjust (13). In this sense, health inequities are systematic differences in health that could be avoided by reasonable means (14). In general, social group differences in health, such as those based on race or religion, are considered health inequities because they reflect an unfair distribution of health risks and resources (3). The key distinction between the terms *inequality* and *inequity* is that the former is simply a dimensional description employed whenever quantities are unequal, while the latter requires passing a moral judgment that the inequality is wrong.

The term health inequality can describe racial/ethnic disparities in US infant mortality rates, which are nearly three times higher for non-Hispanic blacks versus whites (15), as well as the fact that people in their 20s enjoy better health than those in their 60s (3). Of these two examples, only the difference in infant mortality would also be considered a health inequity. Health differences between those in their 20s versus 60s can be considered health inequalities but not health inequities. Health differences based on age are largely unavoidable, and it is difficult to argue that the health differences between younger and older people are unjust, since older people were once younger people and younger people, with some luck, will someday become old.

On the other hand, differences in infant mortality rates among racial/ethnic groups in the United States are partially attributable to preventable differences in education and access to health and prenatal care (15). Unlike the example of age-related health differences, disparities in health outcomes across racial/ethnic groups could be aggressively prevented. Policies and programs that improve access to health and prenatal care for underserved US racial/ethnic groups, for example, could reduce unjust differences in infant health outcomes.

While the existence of health disparities is a near universal problem, the extent to which social factors matter for health has been shown to vary by country. For example, a comparative study of 22 European nations showed that differences in mortality among those with the least versus the most education varied substantially across counties. For example, the authors found less than a twofold difference in mortality between those of high and low education in Spain, and more than a fourfold difference between the two education groups in the Czech Republic (16). Recent evidence suggests that socially patterned health disparities may be widening (17–19), calling for consistent attention to the issues of health inequalities.

There are compelling reasons to worry about, and address, such health differences. The persistence of health differences based on nationality, race/ethnicity, or other social factors raises moral concerns, offending many people's basic notion of fairness and justice (13, 20). Although myriad resources and outcomes are unevenly distributed across nations and social groups, health differences can be viewed as particularly objectionable from a human rights perspective (21, 22). The concept of health as a human right was enshrined in the United Nations General Assembly's Universal Declaration of Human Rights in 1948 (23) and has since been reflected in national constitutions, treaties and domestic laws, policies, and programs in countries around the world (22), emphasizing the unique value societies place on health. Increasingly, health equity itself is also valued. For example, the World Health Organization recognizes health equity as a priority, reflected in part by its formation of the Commission on Social Determinants of Health in 2005. This commission gathers and synthesizes global evidence on social determinants of health and recommends actions that address health inequities (24). Similarly, the United Nations (UN) has also placed an explicit value on equity. The UN's Millennium Development Goals (MDGs), which expire at the end of 2015, have focused on average-based targets that obscure inequalities. In the post-MDG era, the UN has included equity in its post-2015 sustainable development agenda. One of the six ‘essential elements’ that form the core of the post-2015 negotiations focuses on fighting inequality, in part by addressing gender-related health disparities and inequitable access to health care (25).

From a strictly utilitarian standpoint, the cost of health inequalities is staggering. Between 2003 and 2006 alone, the direct economic cost of health inequalities based on race or ethnicity in the United States was estimated at $230 billion. Researchers calculated that medical costs faced by African Americans, Asian Americans, and Hispanics were in excess by 30% due to racial and ethnic health inequalities, including premature death and preventable illnesses which reduced worker productivity. When indirect costs were factored into the calculations, the economic burden was estimated as $1.24 trillion (26). In addition to the costs that could be avoided if socially disadvantaged groups enjoyed equitable health outcomes, inequality itself may be harmful to health. A review of 155 papers that explored income inequality and population health found that health tends to be poorer in less equal societies, especially when inequality is measured at large geographic scales (27).

Whether motivated by economic or moral considerations, the study of, and fight against, health inequalities requires a familiarity with relevant definitions, concepts, and theories of health differences.

## Concepts for operationalizing the study of health inequality

### Group-level differences versus overall health distribution

There are two main approaches to studying inequalities within and between populations. Most commonly, we examine differences in health outcomes at the group level to understand social inequalities in health. For example, we might ask how mean body mass index (BMI) of the poor compares to that of the rich. Because recognizing social group differences in health is necessary for targeting investments to the worst off groups, a group-level approach can support the creation of laws and programs that seek to eliminate social group differences. Because social inequities in health are shaped by unfair distributions of the social determinants of health, tracking social group differences in health is important for monitoring the state of equity in a society. The World Health Organization, for example, recommends that health indicators be reported by groups, or ‘equity stratifiers’ for the purposes of monitoring health inequities (5). Also, focusing on social groups allows us to understand current health inequalities in a historical and cultural context, which provides insights into how health differences may have arisen. For example, considering the history of slavery and segregation in the United States sheds light on current racial/ethnic health disparities. Similarly, understanding the political and religious history of the caste system in India helps us understand how it affects social status, occupation, education levels, and health outcomes for individuals today. In short, viewing health disparities through the lens of social groups can help guide interventions, enable surveillance of important equity issues, and advance our understanding of health by helping us make connections that may have not been initially obvious (3, 6).

Alternatively, it is possible to focus on health differences across individuals, for example, describing the range or variance of a given measure across an entire population. This method is agnostic to social groupings, effectively collapsing all people into one distribution (8). Researchers studying global income inequality have used this approach to highlight the relative wealth of poor individuals in rich countries compared to well-off individuals in poor countries, for example, (28). In contrast to focusing on how people from similar backgrounds compare to one another, exploring the income distribution across one global population has yielded important insights into just how unequally resources are currently distributed, as well as what factors drive these differences.

It can also be useful to compare outcomes across individuals within a single country. For example, applying this approach to the study of inequalities in BMI in India might yield data on the difference in BMI from the fattest to thinnest person. While examining inequalities across individuals provides important information on how outcomes are distributed, it does not allow us to understand who fares better or worse, and whether the gap between the healthy and sick is preventable or unjust. Despite this limitation, some researchers have argued that considering the overall health distribution of a population is especially useful for comparing health in different places because social groups are defined differently, and carry different meanings, across the world (8). For example, race is defined differently in the United States than it is in other countries, while social grouping according to caste is relevant for just a handful of countries, including India, Nepal, Pakistan, and Sri Lanka. Considering the overall health distribution of a population may also avoid making incorrect assumptions about what social groupings matter in a particular place. Despite the challenges associated with measuring and interpreting social inequalities in health, the remainder of this article focuses on health inequalities across social groups rather than individuals.

A critical step in examining group-level health inequalities is defining the relevant social groups themselves. The World Health Organization highlights place of residence, race/ethnicity, occupation, gender, religion, education, SES, and social capital or resources as particularly relevant stratifiers that can be used to define social groups (5). Below we introduce considerations for studying health inequalities that operate across social groups. This section is followed by a discussion on exploring social group differences in health within geographies. With cross-country comparisons of health outcomes regularly reported by international bodies such as the World Health Organization (e.g. 10) and growing interest in within country analyses (e.g. 29), understanding how to approach geographic health inequalities is fundamental for researchers and practitioners.

### Social group health inequalities: defining groups

Health disparities along racial, ethnic, and socioeconomic lines are observed in both low- and high-income countries, and may be widening (9), underscoring the importance of studying of group-level health differences. Understanding socially patterned health disparities requires constructing meaningful groups of individuals. Each society has its own unique ways of stratifying and dividing people into social groups. In Australia, the distinction between white Australians and aboriginal people is meaningful, while in India, caste is important. Race/ethnicity is a particularly meaningful distinction in the United States, while the level of schooling achieved contributes to social divisions in the United Kingdom. We discuss considerations for constructing and interpreting measures of social group health inequalities below.

Researchers and consumers of information on health differences should carefully consider how social groups are constructed, as health inequality data can only be interpreted with respect to group composition. Some social groupings are based on categories of membership, as is in the case with religion or race, while others are created according to ordered or continuous levels of a given variable, such as education or income. Clearly defined membership categories grounded in theory and backed by *a priori* contextual knowledge can facilitate the study of health inequalities, though researchers will have to make decisions about when to collapse or further differentiate groups. For example, should Catholics and Protestants be broadly categorized under the umbrella Christian, or are denominational differences important? Is it meaningful to compare non-Hispanic whites to minorities in general, or does each racial/ethnic group require its own category? Increasingly complex considerations, including, for example, how race and ethnicity are defined, differentiated, and conceptualized (30, 31), add to the challenge of meaningfully comparing social groups. Such questions can only be answered with respect to the specific hypotheses being tested, or the disparities monitored, and should be grounded in context and theory. In general, however, it is important to be aware that group construction will drive the interpretation of health inequality data.

Alternatively, health differences can be patterned with respect to an ordered or continuous quantity such as education or income. Two key questions should be considered in these cases. First, do we believe that health outcomes hinge on meeting some benchmark with regard to the social resource (i.e. a threshold model), or do we predict a social gradient in health that exhibits more of a dose–response relationship? Secondly, do we believe that an individual's response to the social variable depends only on his own level of that variable, or does it matter where he ranks with respect to others?

A ‘social gradient’ in health (32, 33) exists where increasing quantities of social resources such as education, social class, or income correspond with increasing levels of health in a dose–response relationship (see Table 1 for examples). As an example, consider education, which is well known to positively impact health (35). The relationship between education and health is such that even at very high and low ends of the education distribution, additional years of school correspond with marginally better health. If instead of a functioning as social gradient, education had a threshold effect on health, we might observe that not having a secondary school education was associated with worse health but that education and health were not linked for those who had completed secondary school or a higher degree. For example, under this threshold model, we would not expect those with a graduate school education to be healthier than those with a college education. Policy responses to dose–response versus threshold effects of social resources would be quite distinct, and so researchers should be sure to differentiate between the two. Whether a dose–response curve or threshold effect better represents the relationship, studying effects at high and low levels of education is critical. Plotting the relationship between health and education, with education on the x-axis and health on the y-axis, for example, would reveal the shape of a curve describing how additional schooling impacts health. That shape describes how health responds to schooling across the educational spectrum, including whether a threshold exists beyond which education impacts health very little, and the extent to which additional school matters for high and low education individuals.

**Table 1:** Indicators of socioeconomic position used in health research measured at the individual level

Education	Usually used as categorical measuring the levels achieved; also as a continuous variable measuring the total number of years of education
Income	Indicator that, jointly with wealth, directly measures the material resources component of SEP. Usually measured as household gross income per number of persons dependable on this income
Wealth	Includes income and all accumulated material resources
Occupation-based indicators	
The Registrar General's Social Classes^a^	Groupings of occupation based on prestige in six hierarchical groups: I (highest), II, III non-manual, III-manual, IV, V (lowest). Often regrouped as manual versus non-manual
Erikson and Goldthorpe Class Schema	Groupings of occupations based on specific characteristics of employment relations such as type of contractual agreement, independence of work, authority delegation, etc. Not a hierarchical classification
UK National Statistics Socio-Economic Classification^b^	Based on the same principles as the Erikson and Goldthorpe scheme. Creates non-hierarchical groups
Wright's Social Class Scheme	Based on Marxist principle of relation to the means of production. Not a hierarchical classification
Cambridge Social Interaction and Stratification scale	Based on patterns of social interaction in relation to occupational groups
Occupational-based census classification	For example, US census classification, country-specific socioeconomic classifications
Other indicators	
Unemployment	Lack of employment
Housing	Housing tenure, household amenities, housing characteristics, broken window index, social standing of the habitat
Overcrowding	Calculated as the number of persons living in the household per number of rooms available in the house (usually excluding kitchen and bathrooms)
Composite indicators	At individual (usually measured as a score that adds up the presence or absence of several SEP indicators) or at area level
Proxy indicators	These are not strictly indicators of SEP but they can be strongly correlated with SEP and when more appropriate information is not available they may be useful in describing social patterning. Some cases may provide insight into the mechanism that explains the underlying association of SEP and a particular health outcome. However, they may be associated with the health outcome through independent mechanisms not related to their correlation with SEP

a Also known as British Occupational-based Social Class.

b Current official indicator of SEP in the UK, also known as NS-SEC scheme.

Source: Taken directly from Galobardes et al. (34).

### Absolute versus relative social position

The second, related question deals with whether absolute or relative (36) position matters for health. This is particularly important when considering poverty, which can be defined in an absolute sense by comparing a given income to a static benchmark, or in a relative sense by comparing a given income to the overall distribution of incomes in a population (37). Absolute poverty definitions rely on a fixed monetary threshold called a poverty line, though this threshold in generally specific to year, country, and household size. Those with incomes falling below the threshold are considered impoverished. On the other hand, relative poverty is defined by comparing a given income to the distribution of income in a population. For example, those earning less than 30% of the national per capita income might be considered relatively impoverished, meaning that the poverty definition changes as average income increases. Among other distinctions between the two ways of defining poverty, it is important to note that a relative poverty definition may classify a greater proportion of a population as impoverished, especially in countries with high levels of income inequality (3).

Notions of absolute versus relative poverty highlight that measures of income can be both objective and subjective. The amount of money in one's bank account is an objective measure of wealth. Whether someone feels wealthy or poor in relation to his neighbors is a subjective measure of wealth. Absolute poverty, which is an objective measure of wealth, is a useful measure for testing the *absolute income hypothesis*, which posits that an individual's health depends only on his own income and not on what others in a population earn (3). By this logic, the health of an individual whose income stays constant should remain unchanged as those around him become wealthier. Similarly, it would predict that earning $50,000 per year had the same effect on health regardless of whether one's neighbors earned an average of $30,000 or $1 million annually. The absolute income hypothesis ignores the fact that as society becomes wealthier, the material goods needed to fully participate in society can change. Goods such as cars, phones, and computers are now more important than ever to accomplish tasks such as getting to work or accessing health care. As a result, those with static incomes in a changing society may fall behind, potentially suffering psychological distress and stress-related health effects from being unable to keep up with average standards of consumption (3). The relative income hypothesis, which considers subjective measures of wealth, has the advantage of considering psychosocial pathways linking income to health; though testing the hypothesis requires making assumptions about how individuals compare themselves to others. For example, do low-income families feel socially excluded only when other low-income families begin earning more, or do the rising income of celebrities matter as well (3)? It is also possible that relative income matters through other mechanisms as well, with income distribution affecting the ways in which businesses and governments invest in serving the poor (38). Studies that focus on overall income distribution as a determinant of health often use a statistic called the Gini coefficient (39), which summarizes income inequality, to help predict outcomes.

As noted briefly earlier, while the differentiation of relative versus absolute position is particularly relevant when social groups are defined by income, this concept extends to other ordered stratification variables that measure the extent to which individuals are falling behind others around them. These variables may be alternative constructs for measuring access to resources in the place of income, poverty, or wealth measures. For example, Townsend created an index that took account of diet, clothing, housing, work, recreation, and education, among other factors, to measure deprivation in the UK (40). This approach to creating a multidimensional poverty measure has also been utilized to better understand deprivation in the developing country context (41). The distinction between absolute and relative position also matters outside the realm of material or economic deprivation. For example, researchers have examined the impact of winning an Academy Award on all-cause mortality among nominated movie stars in order to investigate whether relative differences in social status mattered for the health of individuals who all uniformly enjoyed high absolute levels of prestige and social status (42). Interest in relative measures of SES, broadly speaking, has grown alongside research arguing that inequality itself harms health (43). Multilevel modeling techniques (44) that allow us to disentangle the influence of individual characteristics from those of higher level structures have also been instrumental in advancing this stream of research into inequality as an independent health risk factor.

### Geographic health inequalities: place versus space

Geographic setting, not just social group, plays an important role in shaping health (45–47). Differentiating the concepts of *space* and *place* helps us to better understand the different ways in which geography can affect health (48). S*pace* deals with measures of distance and proximity such that exposure to spatially distributed health risks and protective factors will change according to an individual's precise location. For example, air pollution that exacerbates asthma symptoms would be an example of a health risk that is distributed across space. Proximity to landfills, crime clusters, and health clinics are other examples of spatially patterned health risks and protective factors. In contrast, *place* refers to membership in political or administrative units, such as school districts, cities, or states. Many government run programs and policies that affect health, such as food assistance programs or tax policies, are specific to administrative units and operate uniformly within their boundaries. As a result, the health impacts of a wide range of programs and policies do not depend on residents’ precise physical location, but rather on membership in a given political or administrative unit.

Concepts of space and place are often treated as exchangeable, and it is easy to see why. Political and administrative units are geographically defined such that people in the same place are often also very close together in space. However, if we imagine an example in which individuals are simultaneously exposed to health risks from a polluting local factory and to health benefits from a village aid program, the conceptual differences become clear. In this example, moving farther from a point source of pollution could improve health, regardless of whether the move were to a location inside or outside the village boundaries. In contrast, maintaining aid would be contingent on residing within village boundaries regardless of where within the village a person lived. Observed geographic health disparities may be driven by processes that are rooted in space, place, or both. From a research standpoint, the studies one might propose to understand geographic health inequalities should account for whether hypothesized health risks are spatial versus place-based. From a policy perspective, programs and interventions could more effectively target geographic health disparities if space and place were both explicitly considered.

### Tracking health inequalities over time

Regardless of how researchers operationalize the study of health inequalities, they also must decide how to report observed differences. Inequalities between groups can be expressed as absolute differences or as relative differences (49, 50). Computing absolute differences involves subtracting one quantity from another, while expressing relative difference requires dividing one quantity by another to produce a ratio. As health differences are tracked over time, absolute differences between groups can increase while relative differences increase, or vice versa. For instance, if 10 people per 100,000 are hospitalized for asthma each year in State A while 20 per 100,000 are hospitalized for asthma in State B, the absolute difference in asthma hospitalizations is 10 per 100,000. There are a few points to note in this example. First, both villages enjoy very low asthma hospitalization rates, though this fact is lost when only reporting on the magnitude of the inequality. Secondly, while a difference of 10 hospitalizations per 100,000 is relatively small, the villages appear to have vastly asthma hospitalization rates when the difference is expressed as a ratio.

As inequalities are tracked over time, decisions about how to express health differences become even more complex. Imagine that we follow our two hypothetical villages for 10 years and find that asthma hospitalization rates have increased in each. Now, 45 per 100,000 are hospitalized in State A while 60 per 100,000 are hospitalized in State B. The new absolute difference has risen to 15 per 100,000, but the relative difference has actually fallen such that State B has only 33% more hospitalizations than State A. In 10 years, asthma hospitalization rates in both states have increased, as has the absolute difference between states. At the same time, relative health inequalities have narrowed. Selective reporting of absolute or relative differences makes it difficult to understand if populations are faring better or worse over time, and by how much. In general, providing baseline information, as well as data on absolute and relative differences, presents a fuller picture of trends in health inequalities.

## Framework for understanding health inequalities

Previous sections of this article dealt with practical issues of how health inequalities can be measured, including whether health differences are studied across individuals or groups, how inequalities may be measured across geographies and social groups, and how observed differences can be reported cross-sectionally and over time. We now move to concepts that are useful in considering how inequalities arise, and for exploring causal mechanisms that link geographic or social group membership to health. These are generic concepts that can apply both to the study of social inequalities in health and to understanding health inequalities across individuals.

### Causal pathways and conditional health effects

When studying the relationship between an exposure, such as occupation, and an outcome, such as blood pressure, it often becomes clear that a third variable matters as well. Variables that lie on the causal pathway between exposure and outcome, called *mediators*, are those that explain how a given exposure leads to an outcome of interest (51). For instance, in a study of occupation and its effects on blood pressure, we might learn that income is the link that explains how a person's job influences their blood pressure. In this example, occupation could determine income, which then might affect blood pressure by influencing whether a person can buy healthy food, receive adequate medical care, or experiences stress over financial matters. When designing policies or programs to influence an outcome like blood pressure, it may be effective to consider ways that income could be used as a policy tool. For example, if income is responsible for the link between occupation and blood pressure, cash transfers or public assistance for low-income workers could improve blood pressure without changing working conditions. However, we might find that, even after increasing income, occupation still has an impact on blood pressure. If this were the case, we would conclude that income only partially mediates the occupation–blood pressure relationship. Knowing that occupation has an effect on blood pressure independent of income might spur researchers to ask whether job stress or working conditions affect health. Studies of health disparities should try to identify these pathways whenever possible because doing so helps us to better understand the mechanisms by which health differences arise and provides more options for designing policy solutions to real-world problems.

Key Terms:

Mediator: A variable that lies on the causal pathway between exposure and outcome, helping to explain the association between them.

Effect modifier: A variable that does not lay on the casual pathway between exposure and outcome, but whose presence helps explain when and how an exposure and outcome are related. The relationship between exposure and outcome may vary according to the level of the effect modifier.

In other cases, we may discover that a third variable, often called a modifier or moderator, helps explain the conditions under which an exposure and outcome are related (51). Returning to the example of occupation and blood pressure, we can consider the role of race in the workplace. In many contexts, racial discrimination persists in the workplace. Within such a context, white employees who receive promotions might experience a decrease in blood pressure, perhaps due to increased job control and workplace status. On the other hand, black employees might not reap any health benefit from promotions because discrimination persists at all occupational levels, preventing them from feeling a sense of increased status or control at work. In this example, we might observe that better occupations improve blood pressure for white, but not for black, employees. Unlike our first example, in which income had a clear, directional impact on blood pressure, our second example shows how race modifies the relationship between occupation and blood pressure in different ways. This example also reminds us that social groups are not simply of interest as exposures, but may also explain the relationship between other exposures and outcomes.

### Selection

*Selection* is another fundamental concept for understanding health inequalities (52). Selection refers to the fact that people have a tendency to sort themselves into neighborhoods, social groups, and other clusters. For example, people who value physical activity may be more likely to move to walkable areas, while sedentary individuals might choose to live in auto-dependent suburbs. When we see data suggesting that neighborhood walkability affects whether residents are physically active, therefore, we have to ask to what extent the observed relationship is causal, and to what extent it simply reflects self-selection into neighborhoods.

Selection is also sometimes proposed as an explanation for educational, occupational, and even racial/ethnic differences in health. For example, some might attempt to explain the relationship between SES and health as a product of selection by arguing that genetically superior individuals are more likely to have good health and high IQ, therefore explaining why highly educated, high income individuals are generally healthier. Research studies designed to estimate the causal effects of social factors on health generally reject such explanations, however, showing that exposures such as occupation, income, discrimination, and neighborhood poverty, for example, do influence health (35).

### Context versus composition

When selection may be a source of geographic health inequalities, researchers generally want to distinguish *contextual* from *compositional* effects (53). *Contextual* effects refer to the influence a neighborhood or other type of higher level unit has on people, while *compositional* effects are simply reflective of the characteristics of individuals comprised by the neighborhood or other setting. Classrooms, schools, neighborhoods, states, hospitals, and other units of organization can all exert contextual effects. Contextual factors that affect health include policies, infrastructural resources, and public support programs (3) and are, therefore, potential targets of intervention for reducing health inequalities.

Compositional effects refer to variations in health attributable to the health status of the individuals who are members in a given context. If the construction of a specialized healthcare facility suddenly attracted large numbers of chronically ill residents to a given neighborhood, the poor health status of residents in that neighborhood compared to surrounding areas would be compositional.

Differentiating compositional versus contextual effects is of primary importance for making causal inferences about how settings impact health. Knowing that health inequalities exist across contexts does not tell us anything about why differences exist: Does living in high poverty neighborhoods increase the risk of getting sick? After taking individual-level risk factors into account, are there still variations in health outcomes across high and low poverty neighborhoods? Furthermore, does neighborhood poverty have the same health impact on all social groups, or are some at particular risk? Concentrated poverty and many other contextual characteristics may not just impact the average health of a community, but also health disparities between social groups (3).

### Life course perspective

The impact of geography and social group membership on health is not only powerful but also persistent. Differences in early life and *in utero* circumstances can impact later health regardless of subsequent life events, generating health inequalities between social groups. (54, 55). There are critical or sensitive developmental periods during which health is affected in ways that cannot be completely reversed. For example, poor nutrition in adolescence, when bones develop, could put individuals at risk for bone fracture in later life, regardless of attempts to slow bone loss in adulthood. Habits that develop early in life may influence the trajectory of one's health choices. Poor exercise habits in childhood may influence the choices that people later make as adults. Although adults can choose to exercise more later in life, childhood habits may serve as predictors of adult choices that continue to impact health. Finally, long-term exposure to conditions over the course of a lifetime also affects health. Earning a low income may have a greater effect on individuals who grew up poor than for those who grew up rich, for example. This prolonged deprivation could amplify the health effects of poverty.

Key Terms (56):

Life course perspective: A consideration of health inequalities that acknowledges that one's health status reflects both prior and contemporary conditions, including *in utero* and childhood effects. The life course perspective recognizes the impact of latent, pathway, and cumulative effects on later health.

Latent effects: Health effects caused by prior conditions that impact later health, regardless of subsequent life events. Examples include lack of adequate prenatal care or poor nutrition in childhood.

Pathway effects: Health effects resulting from early life conditions, which continue to impact future behavior. Examples include poor exercise habits in childhood that continue into adulthood. Although these habits can be changed in adulthood, they can be predictors of adult choices that themselves have health effects.

Cumulative effects: Health effects resulting from long-term exposure to conditions that affect health. Examples include prolonged exposure to environmental toxins or long-term poverty.

**Fig. 1:**
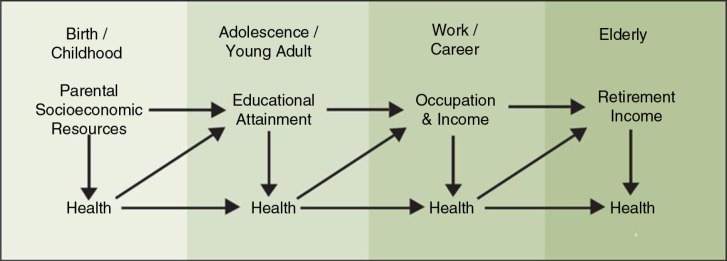
The impact of socioeconomic status on health across the life course. Source: Taken directly from Adler et al. (57).

When social mobility is low and socially marginalized groups have historically limited options about where to live, early life conditions may be especially powerful in explaining current health inequalities. For example, in societies that struggle with the intergenerational transfer of poverty, or have a long history of ghettoizing marginalized groups, it is likely that individuals currently exposed to socially patterned health risks were previously exposed to socially patterned health risks as well, see Fig. 1 (57). Researchers should be aware that lagged exposures, even those as distant as parental occupation or childhood neighborhood, may be useful in explaining current health outcomes. Subject matter expertise in human development should inform studies or projects that explore prior life conditions to explain current health differences between groups. Longitudinal data, in addition to allowing for the exploration of lagged or cumulative effects, are also crucial for understanding the direction of causal relationships driving associations between health and social conditions. For example, recent evidence suggests that neighborhood poverty may indeed increase health risks (58), but that poor health may also systematically sort individuals into poorer neighborhoods (59). Only longitudinal study designs can help to clarify whether and the extent to which challenging social conditions and poor health outcomes reinforce each other over time.

## Explaining health inequalities

Social epidemiologists apply the concepts presented above to help measure and understand health inequalities. Several broad categories of explanations (3, 54, 60, 61) are generally tested when trying to explain health differences across geographies and social groups but may also drive health differences across individuals in a population.

One type of explanation points to *material* factors in the creation of health disparities. Material factors include food, shelter, pollution, and other physical risks and resources that influence health outcomes. Measures of absolute resources, such as absolute income, are useful in testing the role of material deprivation in creating health differences, as are objective measures of physical health risk factors such as air quality. An unequal distribution of physical health risks and resources across geographies and social groups contributes to social inequalities in health via material pathways.

A second class of explanation points to *psychosocial* (62) factors as driving health inequalities and social group differences in health in particular. Psychosocial health impacts stem from feelings of social exclusion, discrimination, stress, low social support, and other psychological reactions to social experiences. Negative psychological states affect physical health by activating the biological stress response, which can lead to increased inflammation, elevated heart rates, and blood pressure, among other outcomes (63, 64). Measures of relative position, perceived versus objectively measured variables, and instruments that capture different experiences of stress are all useful in studies of psychosocial risk factors. To the extent that certain social groups are systematically more likely to have stressful, demoralizing, and otherwise emotionally negative experiences, psychosocial factors can help explain health inequities.

*Behavioral* differences are also commonly cited as contributing to health inequalities. For example, a behavioral explanation might attribute health inequalities to differences in eating habits, smoking prevalence, or cancer screening rates across social groups or across individuals in a population. While health behaviors often do vary across groups, ecosocial (65, 66) and social–ecological (67) frameworks prompt us to ask what upstream factors might be responsible for these variations. For example, if differences in smoking rates are caused by unequal educational opportunities, an inequitable distribution of psychosocial risk factors, and targeted marketing, attributing health disparities to behaviors may be of limited usefulness.

A fourth type of explanation points to differences in biological health risk factors that are patterned across social groups or contexts (60, 68), or vary across individuals in a population. *Biomedical* explanations can suffer the same weaknesses as behavioral explanations for social inequalities in health when they focus on the downstream effects of social context without acknowledging why levels of biological risk factors vary across populations. Genetic and gene-by-environment interactions explanations are also, in part, biomedical in their nature. This class of explanation may be more useful for understanding variations in health observed across individuals in a population where social group differences are not the focus of investigation.

Applying a life course perspective to the consideration of all four types of explanations while considering that factors from each category may be main exposures, mediators, or moderators creates useful complexity in thinking about how health inequalities arise.

## Conclusions

This article has introduced definitions and concepts that may be combined and applied in a wide range of settings. Previous work on health inequalities has introduced critical concepts and explored defining questions (3), evaluated relevant theories and considered resulting policy implications (4), discussed measuring and monitoring disparities (5, 7, 69), among other contributions. Building on these and other valuable resources, this paper has sought to unite salient theories, concepts, and methods into a single article, and to highlight previously under-discussed aspects of disparities research, such as the distinctions between space and place. When considering differences in health, it is important to determine whether inequalities were measured across individuals in a single population, or describe group-level differences. Group definitions will vary by historic and social context, and establishing meaningful groupings will be specific to those contexts. Social group health inequalities may be generated early or late in life by differences in access to material resources, social circumstances that generate stress, or health behaviors. Understanding causal pathways linking social factors to health, as well as conditional health, can aid in intervention planning. Geographic health disparities are also common and often reflect unjust social structures. Differentiating the concepts of place and space can help uncover what generates geographic health differences.

Even more difficult than executing well-designed studies of health inequalities is deciding what to study and how to use findings to narrow gaps between groups. A central task is deciding when a health inequality is inequitable, and why. Setting a policy agenda around health inequities is also fraught with difficult questions and decisions, including whether it is better to reduce absolute or relative health differences between groups; whether to focus on improving health for the worst-off groups or for the largest groups; and how to set benchmarks for health outcomes for various groups. For example, should we set the target life expectancy for black Americans to that of whites, or should we be aiming for both groups to live even longer? Are certain social groups or health outcomes more deserving of attention than others? If so, why? Do particularly unjust health differences deserve attention, or should we focus on health outcomes that are especially expensive or prevalent? What are the merits of investing resources into improving overall population health, and what are arguments for focusing on the elimination of health disparities instead?

There are no clear cut answers to any of these questions, though they are among the central factors shaping how health inequalities are studied and discussed. Criteria for prioritizing scarce resources may by economic, political, moral, or practical. These and other factors must be weighed in crafting research and policy agendas to track and understand health inequalities.
